# Assessing the Chemical Intelligence of Large Language Models

**DOI:** 10.1021/acs.jcim.5c02145

**Published:** 2025-12-18

**Authors:** Nicholas T. Runcie, Charlotte M. Deane, Fergus Imrie

**Affiliations:** Department of Statistics, University of Oxford, Oxford OX1 3LB, U.K.

## Abstract

Large Language Models are versatile, general-purpose tools with a wide range of applications. Recently, the advent of “reasoning models” has led to substantial improvements in their abilities in advanced problem-solving domains such as mathematics and software engineering. In this work, we assessed the ability of reasoning models to perform chemistry tasks directly, without any assistance from external tools. We created a novel benchmark, called ChemIQ, consisting of 816 questions assessing core concepts in organic chemistry, focused on molecular comprehension and chemical reasoning. Unlike previous benchmarks, which primarily use multiple choice formats, our approach requires models to construct short-answer responses, more closely reflecting real-world applications. The reasoning models, OpenAI’s o3-mini, Google’s Gemini 2.5 Pro, and DeepSeek R1, answered 50%–57% of questions correctly in their highest reasoning modes, with higher reasoning levels significantly increasing performance on all tasks. These models substantially outperformed the nonreasoning models which achieved only 3%–7% accuracy. We found that Large Language Models can now convert SMILES strings to IUPAC names, a task earlier models were unable to perform. Additionally, we show that the latest reasoning models can elucidate structures from 1D and 2D ^1^H and ^13^C NMR data, with Gemini 2.5 Pro correctly generating SMILES strings for around 90% of molecules containing up to 10 heavy atoms, and in one case solving a structure comprising 25 heavy atoms. For each task, we found evidence that the reasoning process mirrors that of a human chemist. Our results demonstrate that the latest reasoning models are becoming increasingly capable of performing advanced chemical reasoning.

## Graphical abstract

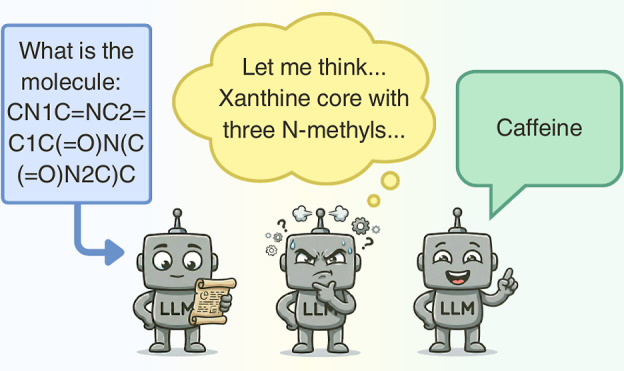

## 1 Introduction

Large language models (LLMs) have emerged as powerful general-purpose tools across a variety of domains.^1^ Trained on extensive corpora of natural language, these models initially excelled at generating coherent text but often struggled with complex tasks that required deeper problem solving and reasoning. This limitation was at least partially addressed with the introduction of Chain-of-Thought (CoT) prompting, where the model is encouraged to conduct intermediate reasoning steps before arriving at a final answer.^2,3^ This has recently led to the development of “reasoning models” which are explicitly trained to optimize their CoT across a range of tasks.^4^ By training LLMs with reinforcement learning, these models are able to develop reasoning strategies that are broadly applicable across multiple domains. Several reasoning models have been released recently, including OpenAI’s o-series models,^5,6^ DeepSeek R1,^4^ Google’s Gemini 2.5 Flash and Pro,^7,8^ and Anthropic’s Claude 3.7 Sonnet.^9^ These reasoning models have achieved substantial improvements on various benchmarks in mathematics, science, and coding.^4,5,8^

The success of LLMs has prompted investigation into whether these models can aid scientific discovery by performing tasks such as generating novel hypotheses, planning experiments, and interpreting data.^10,11^ LLMs are a potential collaborative tool for chemists and, if their performance continues to improve, may themselves lead to the discovery of novel chemical matter such as new materials, catalysts, and drugs.^12^ There has been some initial exploration of the use of LLMs in chemistry,^13^ with previous studies suggesting that LLMs have a broad understanding of chemistry^14–20^ (see Supporting Information Section A for further details).

However, it has repeatedly been observed that LLMs struggle to interpret molecular structures, thus restricting their wider use in real-world scenarios.^13,21,22^ Researchers have primarily attempted to address this limitation through the development of “agentic systems” where LLMs are given access to external chemistry software to solve tasks that they can not directly perform.^10,13,22,23^ While these systems are able to plan and execute multistep workflows based on user-specified criteria, the developed workflows are typically not designed according to specific molecular structures of interest. LLMs that can directly interpret molecular structures would substantially advance the capabilities of both standalone LLMs and LLM-based systems.

In this work, we investigated the ability of LLMs to autonomously perform a range of chemistry tasks that require molecular understanding, without assistance from external tools. To do so, we have constructed a new corpus of tasks, called “ChemIQ”, specifically to test LLMs’ understanding of organic molecules. Previous LLM benchmarks for chemistry are summarized in Supporting Information A; ChemIQ is distinct from these in three key aspects:

1. The tasks included in our benchmark are highly focused on molecular comprehension, as opposed to previous benchmarks which combine questions from numerous chemistry disciplines. To the best of our knowledge, ChemIQ is the first benchmark to assess LLMs on molecular interpretation tasks such as identifying the shortest path between two atoms, mapping atoms between two representations of the same molecule, performing 2D NMR elucidation, and Free-Wilson analysis. Additionally, we introduce the use of the OPSIN parsing tool^24^ to interpret IUPAC names generated by LLMs, providing robustness to valid but nonpreferred names.
2. All questions are algorithmically generated, allowing new questions to be readily produced. This allows failure modes of LLMs to be probed and benchmarks to be updated, for example by adding more complex questions as LLM capabilities increase, or periodically with new questions to ensure performance is not inflated due to data leakage.
3. ChemIQ more closely reflects potential use cases of LLMs than previous benchmarks. Most previous benchmarks contain exclusively^15,16^ or an overwhelming majority^14^ of multiple choice questions. While these are valid for testing general knowledge, they can often be solved by elimination rather than direct reasoning. ChemIQ consists solely of short-answer questions, requiring the model to construct a solution as opposed to selecting from multiple options. As such, it more accurately reflects real-world tasks and should therefore be a more useful measure of performance.^25^

Using our ChemIQ benchmark, we find that the latest reasoning models now possess the ability to understand molecular structures and directly perform tasks requiring advanced chemical reasoning. Across 816 questions, the state-of-the-art reasoning models, o3-mini, Gemini 2.5 Pro, and DeepSeek R1, answered 50–57% of questions correctly in their highest reasoning level, with the overall accuracy depending on the level of reasoning used. This stands in stark contrast to the nonreasoning models, ChatGPT-4o, Gemini 2.5 Flash (in nonthinking mode), and DeepSeek V3, which answered only 3–7% of questions correctly. For each task, we found similarities between the model’s reasoning process and that of a human chemist, suggesting a level of conceptual understanding of chemistry. Our results demonstrate that the latest reasoning models now have advanced chemical reasoning capabilities and are able to perform tasks that previously relied on expert human judgment.

## 2 Construction of the ChemIQ Benchmark

ChemIQ is a novel benchmark developed to assess the chemical intelligence and understanding of LLMs. We designed the questions in ChemIQ to test the understanding of molecular structures and application of chemical reasoning as opposed to assessing general chemical knowledge or superficial pattern recognition. ChemIQ focuses on three broad competencies: (1) interpreting molecular structures, (2) translating molecular structures to chemical concepts, and (3) reasoning about molecules using chemical theory. Competencies (1) and (2) represent foundational skills necessary for molecular comprehension, while competency (3) assesses more advanced chemical reasoning capabilities. We algorithmically generated a total of 816 questions across eight distinct categories, which are summarized in Figure 1. Unless otherwise stated, we used molecules from the ZINC data set^26^ as examples of drug-like molecules. We represented molecules using Simplified Molecular Input Line Entry System (SMILES)^27^ as this format is widely used in cheminformatics and is a straightforward way for a human chemist to interact with LLM systems. As model performance can depend on prompt design, we deliberately used simple, unoptimized prompts to reflect how a typical chemist might interact with these models and to avoid biasing results toward any particular LLM. Examples of full prompts and corresponding reasoning excerpts generated by o3-mini are provided in Supporting Information D.

**Figure 1:**
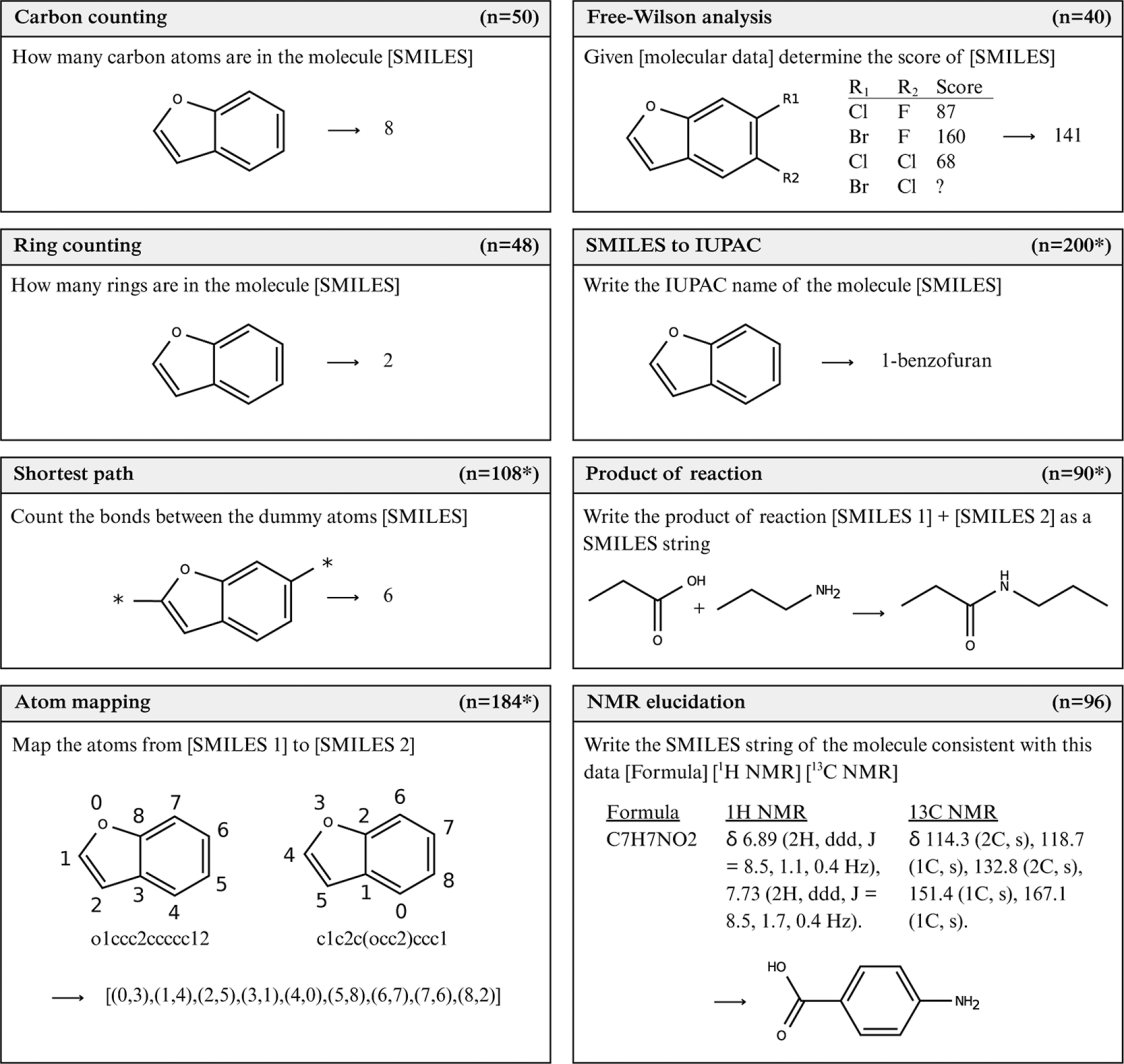
Question categories in the ChemIQ benchmark. The number of questions in each category is shown in the panel header, and * indicates that the set contains 50% canonical and 50% randomized SMILES. Full prompts and example reasoning excerpts can be found in Supporting Information D.

**Table 1:**
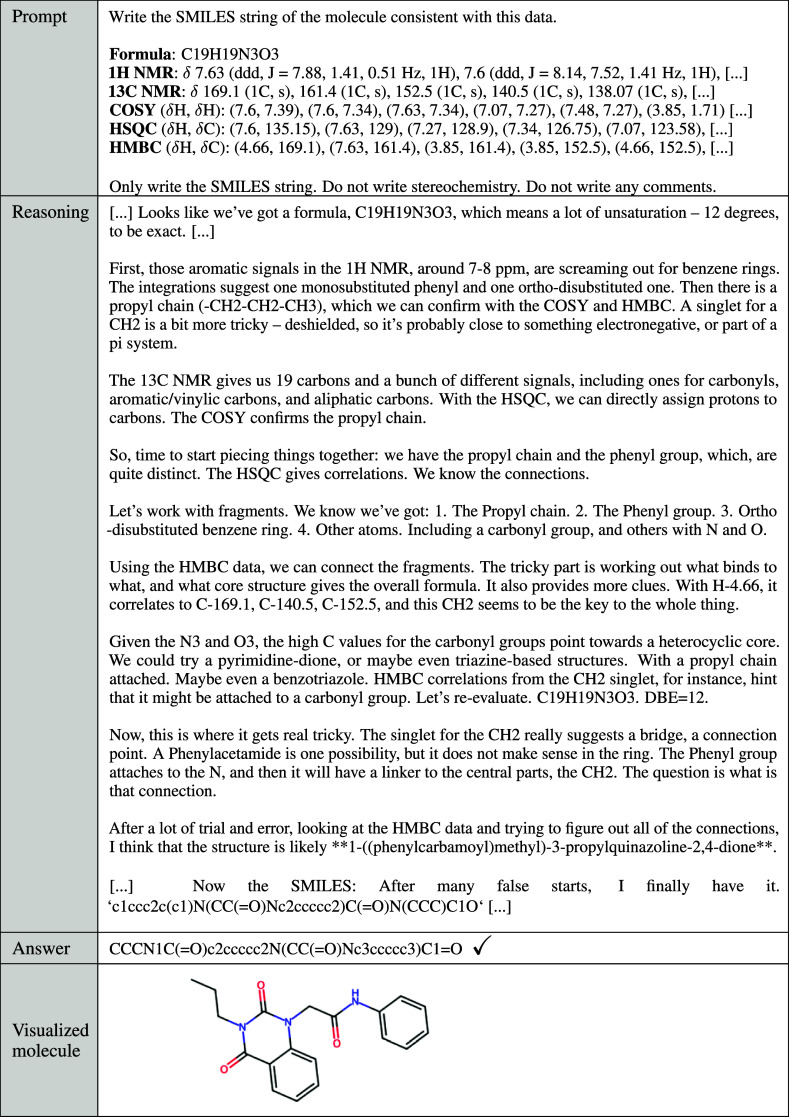
Excerpts of Reasoning Generated by Gemini 2.5 Pro on a 2D NMR Structure Elucidation Question^a^

a The full prompt can be found in Supporting Information S20. The model shows reasoning consistent with that of a human chemist. It combines information from the molecular formula and each NMR spectrum, considering factors such as chemical shifts, multiplicity, double bond equivalents, and cross peaks to deduce the structure of the molecule.

### 2.1 Interpreting Molecular Structures

To demonstrate even a basic understanding of molecules, the model must be able to count. For example, counting the number of atoms of each element is needed to determine the molecular formula. LLMs have previously been known to struggle with counting characters in text,^28^ suggesting potential challenges for interpreting SMILES strings. To test this ability, we asked the LLMs to count the number of carbon atoms and the number of rings in a molecule, properties that can be easily inferred from SMILES notation.

A core skill for molecular interpretation is the ability to extract graph-based features from SMILES strings. Certain chemical features can be identified through simple pattern recognition (e.g., C­(=O)­NC indicates an amide group). Consequently, questions asking for the identification of functional groups within a molecule can give a misleading impression of molecular comprehension. To test the general ability to extract graph-based features from SMILES strings, we created a task that requires the LLM to determine the number of bonds on the shortest path between two randomly selected positions in a molecule. This requires a greater understanding of molecular structure than subgroup identification tasks.

Finally, we also constructed an “atom mapping” task to test global understanding of molecular structures. In this question, we assess whether the model can form an internal representation of molecules and recognize graph isomorphism. While a canonical SMILES format exists, a molecule can be represented by multiple SMILES strings. Furthermore, each atom can be assigned a unique index based on its position within a SMILES string. The LLM was given two randomized SMILES strings of the same molecule and was asked to provide the mapping of atoms from one representation to the other. Successfully answering this question requires an understanding of SMILES strings and molecular graphs, signaling the potential to perform more advanced reasoning on these structures.

### 2.2 Translating Molecular Structures to Chemical Concepts

A key objective for LLMs is to bridge the gap between computational representations of molecules and higher-level chemical terminology. Successfully doing so is necessary for any reasoning task that requires applying chemistry theory to molecular graphs, i.e. describing electronic effects, designing molecules, and performing structure elucidation.

The International Union of Pure and Applied Chemistry (IUPAC) provides a standardized naming convention where molecules are described using common chemistry language; the ability to write the IUPAC names of molecules requires an understanding of molecular structures and their chemical features. Converting SMILES strings to IUPAC names has proven extremely challenging for LLMs, with most models achieving near-zero accuracy.^18,19,29,30^

Previous benchmarks have typically assessed accuracy by comparing generated names to the exact name contained in PubChem,^31^ which itself uses the OpenEye tool “Lexichem”^32^ to determine IUPAC names. While this approach is valid, we believe it is overly strict. Importantly, a single molecule can be identified by multiple valid IUPAC names, not only the one considered as “standard”. While these nonstandard names ignore conventions related to group priority, they nonetheless reflect an equivalent understanding of molecular structure. Therefore, we propose a modification of the SMILES to IUPAC task where a name is considered correct if it can be parsed to the intended structure using the Open Parser for Systematic IUPAC Nomenclature (OPSIN) tool.^24^ OPSIN is widely accepted as a reliable IUPAC name parser (c. 99.8% precision, 99% recall),^24^ and in our benchmark it successfully parsed all molecules (for which we had reference IUPAC names) to the intended structures, although some edge-case failures have been documented.^33^

### 2.3 Chemical Reasoning

For an LLM to be used for novel chemical discovery, it must be capable of interpreting chemical trends in data. We assessed the potential to conduct such analysis by algorithmically constructing a series of Free-Wilson analysis questions. In each case, the LLM is provided data for seven molecules with different combinations of R-groups at three positions of a shared scaffold and is tasked with predicting the value for an unseen molecule. Further details of the scaffold and R-groups can be found in Supporting Information B. This requires the LLM to be able to understand the differences between molecules, attribute values to these differences, and apply this knowledge to unseen examples.

We further tested chemical proficiency using a series of reaction prediction questions. These questions were constructed from nine common reaction classes, such as the copper-catalyzed click reaction and the Simmons-Smith cyclopropanation reaction. In each case, the LLM was prompted to write the product as a SMILES string. While the chosen templates reflect straightforward and unambiguous chemical reactions, correctly answering the reaction prediction questions requires substantial levels of chemical understanding and reasoning.

Last, we assessed the ability of LLMs to interpret multiple sources of chemical evidence through the task of nuclear magnetic resonance (NMR) structure elucidation. While several machine learning models can provide helpful insights for this task, no current method can reliably interpret NMR spectra without expert validation.^34^ We provided the LLM with simulated NMR data, along with a molecular formula, and then prompted the model to generate the corresponding SMILES string. We assessed performance on molecules of up to ten heavy atoms from Huang et al. using only 1D ^1^H and ^13^C NMR.^35^ We additionally assessed the models using larger molecules of up to 30 heavy atoms from ZINC, providing both 1D ^1^H and ^13^C NMR, as well as 2D COSY, HSQC, and HMBC NMR spectra. This task requires pattern recognition in NMR data, mapping these patterns to molecular features, and subsequently constructing a coherent and complete molecular structure consistent with all sources of information.

## 3 Results and Discussion

### 3.1 Experimental Setup

We evaluated the performance of multiple LLMs on our benchmark set of questions, ChemIQ, to assess their ability to understand molecular structures and perform chemical reasoning. A key aim of our experiments was to assess the impact of “reasoning” on these capabilities. For many models, the extent of reasoning can be controlled using a “reasoning budget” parameter. We therefore chose to run o3-mini (available in “low”, “medium”, and “high” reasoning modes) and Gemini 2.5 Pro (with reasoning budgets between 128 and 32,768 tokens). We also ran Gemini 2.5 Flash which showed similar trends to Gemini 2.5 Pro, but with weaker overall performance; the results of this model are listed in Table S5, but are not discussed further. We additionally ran DeepSeek-V3-0324 (nonreasoning) and DeepSeek-R1-0528 (reasoning) as examples of open-source models, and GPT-4o as a nonreasoning baseline for OpenAI models. All prompts were submitted without access to external tools (such as Internet search or code interpreters), unless otherwise stated, allowing us to assess the capabilities of the underlying models. By comparing the same model across multiple reasoning budgets, we can isolate the improvement gained from additional reasoning.

To help understand how the models arrived at their answers, we examined the “reasoning excerpts” generated by the models. Of the models tested, DeepSeek R1 is the only model that provides a raw CoT; the other models do not disclose the raw CoT and instead provide a summary of their reasoning. While these reasoning traces do not necessarily provide a faithful description of the model’s internal computation,^36^ we believe they remain informative. The generation of (additional) reasoning tokens improved performance across all tasks, indicating they are functionally useful. Furthermore, qualitative analysis of the reasoning traces suggests that the models are capable of expressing detailed, chemically coherent, multistep reasoning. A summary of each task is provided in Figure 1, full prompts along with reasoning excerpts from o3-mini can be found in Supporting Information D, and an example 2D NMR elucidation excerpt from Gemini 2.5 Pro can be found in Table 1. All benchmark questions in ChemIQ, answer checking scripts, and LLM outputs, including reasoning traces and excerpts, are available at https://github.com/oxpig/ChemIQ.

### 3.2 Reasoning Unlocks Chemical Intelligence

The reasoning models substantially outperformed the nonreasoning models across the 816 questions in our benchmark. The reasoning models correctly answered 50–57% of questions when run in the highest reasoning mode, whereas the nonreasoning models scored 3–7%. Moreover, increased levels of reasoning enhanced performance across all tasks (Figure 2). However, minimal performance improvement was observed for Gemini 2.5 Pro at the three highest reasoning budgets, suggesting the model was unable to effectively utilize reasoning tokens beyond a certain threshold (Table S3). We observed several model limitations beyond quantitative benchmark performance, discussed in Section 3.10.

**Figure 2:**
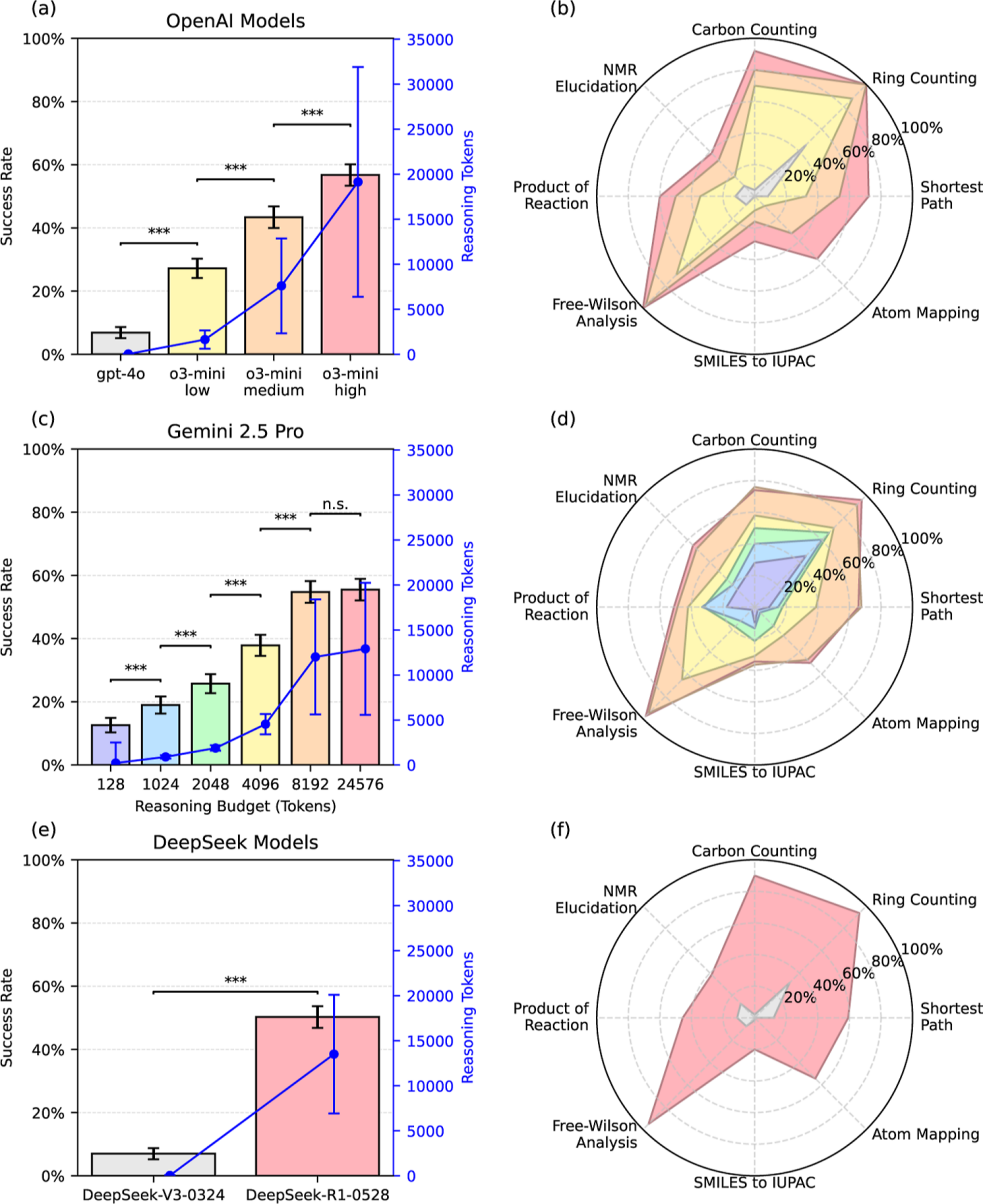
Summary of model performance. Results show a trend that increased reasoning correlates with higher success rates. A summary of question prompts is provided in Figure 1, and full prompts with reasoning excerpts are listed in Supporting Information D. (a,c,e) Average success rates across all benchmark questions and the average number of reasoning tokens used per question. Vertical error bars show 95% confidence intervals (±1.96 × SE of the binomial proportion). Adjacent bars were compared using a one-tailed McNemar test. Significance levels are denoted as *p < 0.05, **p < 0.01, and ***p < 0.001. (b,d,f) Radar plots showing performance by question category. (a,b) OpenAI models GPT-4o (nonreasoning) and o3-mini (reasoning) (c,d) Google Gemini 2.5 Pro with varying reasoning budgets (e,f) DeepSeek models DeepSeek-V3-0324 (nonreasoning) and DeepSeek-R1-0528 (reasoning).

Due to the stochastic nature of LLM outputs, we repeated the ChemIQ benchmark using the OpenAI models five times. Aggregate performance was robust, with limited deviation across repeats for all models (Figure S3a). Performance across each question category showed slightly higher variance, but the overall trends remained consistent (Figure S3b–i). As expected, at the individual question level, there was more variation between repeats: o3-mini-high was the most consistent, answering 33% of questions correctly in every run, 29% never correctly, and 38% of questions correctly in between one and four runs (Figure S5).

The reasoning models answered the majority of numerical tasks involving counting and calculation correctly and demonstrated an ability to interpret graph-based features of molecules. On the SMILES to IUPAC task, the reasoning models achieved 29–44% accuracy on molecules sampled from ZINC; to our knowledge, these are the first general-purpose LLMs to demonstrate meaningful success on this task. Additionally, all reasoning models solved the 1D NMR spectra of molecules up to 10 heavy atoms with 73–94% accuracy, compared with 20–30% accuracy of the nonreasoning models. The Gemini 2.5 Pro model exhibited the best performance in 2D NMR elucidation, correctly solving the structure of ten of the 50 molecules sampled from ZINC, one of which contained 25 heavy atoms.

These results challenge the established view that LLMs cannot comprehend or write molecules in SMILES notation.^18,22,37^ While the performance of the nonreasoning models is consistent with the conclusions from previous studies, the reasoning models can perform tasks that require comprehensive molecular and chemical understanding. However, across our entire benchmark, the best-performing model only successfully answered 57% of questions. Thus, while the latest reasoning models show early signs of being able to perform chemistry tasks, they are not yet able to do so reliably.

In the following sections, we focus primarily on the OpenAI models; however, similar trends were seen for all other models.

### 3.3 Reasoning Models Understand Molecular Structures

A fundamental requirement for understanding organic chemistry is the ability to interpret molecular structures. In this section, we discuss assessing molecular comprehension using a series of tasks that increase in complexity. As an initial test, we examined the ability of models to count characters in the SMILES string, which is a prerequisite for any other task involving the interpretation of SMILES notation. When tasked with carbon counting, GPT-4o only answered 4% of questions correctly. The o3-mini model substantially improved on this, however was still imperfect, scoring between 70% and 92%. Performance on the ring counting task was higher, likely because the sampled molecules contained no more than six rings, which increased the chance of correct guesses and reduced overall task difficulty. These results are consistent with previous observations that LLMs struggle to count characters in text.^38^ Failure at this basic task, even on a small number of cases, may limit the usefulness of SMILES as a chemical representation for current LLMs.

We then examined the ability to understand molecular structures from SMILES strings using more complex questions beyond simple functional groups identification. The reasoning models demonstrated the ability to determine the shortest path between two atoms in a molecule (Figure 3a), with o3-mini-high correctly answering 89% of questions when molecules were written using canonical SMILES, and successfully reasoning about paths of up to 20 bonds. The nonreasoning GPT-4o model only scored 11%. A challenging question is shown in Table S14 where the shortest path crossed a fused ring system. The reasoning excerpt is consistent with o3-mini-high having constructed a graph representation and performed a graph search to solve this question, suggesting the model can parse a SMILES string, build an internal graph representation, and then reason on it.

**Figure 3:**
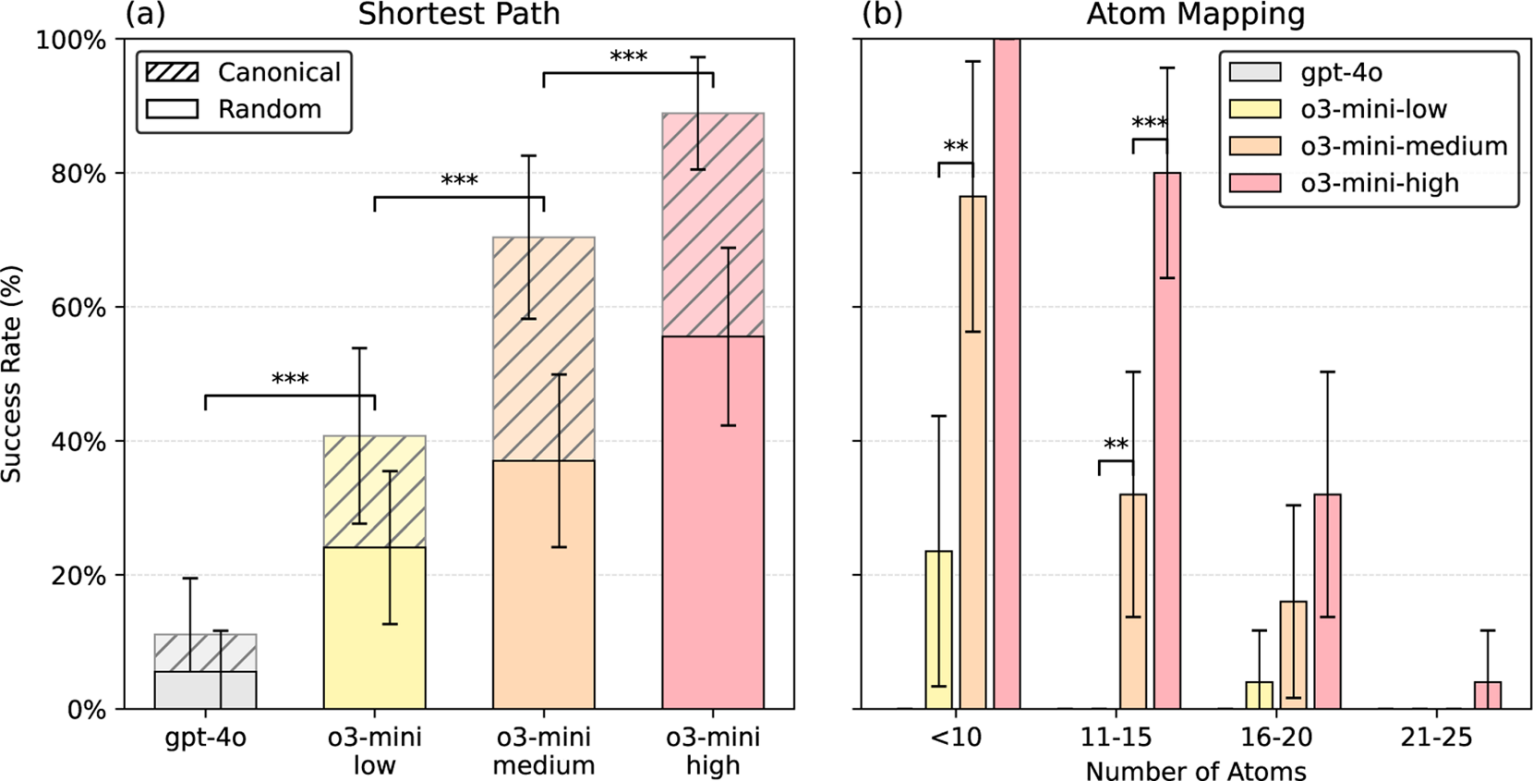
(a) Models were prompted to find the shortest distance between two atoms in a SMILES string. Performance was substantially lower when prompted with random SMILES as opposed to canonical SMILES. (b) Models were given two randomized SMILES strings representing the same molecule, and were prompted to map the atoms from one to the other. Performance decreased for larger molecules. Vertical error bars show 95% confidence intervals (±1.96 × SE of the binomial proportion). Adjacent bars were compared using a one-tailed McNemar test. Significance levels are denoted as *p < 0.05, **p < 0.01, and ***p < 0.001.

Similarly, the reasoning models were able to answer “atom mapping” questions, where the same molecule was written as two different SMILES strings and the model was prompted to map the atoms from one to the other (Figure 3b). The o3-mini-high model answered 56% of questions correctly and successfully mapped molecules with up to 24 atoms, whereas GPT-4o answered none of the questions correctly. As expected, the performance decreased as the number of atoms in the molecule increased, reflecting the greater complexity of atom mapping for larger molecules (Figure 3B). However, the trend in success rate with increased reasoning suggests the ability to answer this question for larger molecules is not an inherent limitation of reasoning models, and may be improved further by using more reasoning tokens.

An example reasoning excerpt for atom mapping is shown in Table S15. The excerpt suggests o3-mini-high used multiple layers of abstraction to solve this question, starting from a coarse-grained description of the molecule before focusing on individual atoms and specifically anchoring on distinctive heteroatoms to aid the search. This demonstrates that reasoning models have the capacity to interpret SMILES strings and navigate molecular graphs; however, this ability is not yet consistent or reliable. Furthermore, the low accuracy on structures larger than 20 heavy atoms implies that current models may face challenges in interpreting typical drug-sized molecules.

### 3.4 Randomized SMILES are More Challenging than Canonical SMILES

SMILES strings can be written in multiple forms depending on the atom ordering used to traverse the molecular graph.^39^ If a model can reliably parse SMILES strings, the atom numbering should not impact the ability to interpret the molecular graph. We repeated the shortest path, atom mapping, SMILES to IUPAC, and reaction prediction questions using canonical and randomized SMILES, and found the reasoning models generally had lower performance when prompted with randomized SMILES (Table S2).

This effect was most prominent for the shortest path question, where the performance of o3-mini, at each reasoning level, dropped by approximately 40% (Figure 3a). This question, in particular, is made more challenging by considering randomized SMILES. Canonical SMILES strings are always written starting on a dummy atom, meaning the shortest path can often be inferred by the position of the second dummy atom in the string (together with consideration of branching and ring closures). In contrast, in randomized SMILES, the string-based distance between the dummy atoms typically differs significantly from the graph-based distance.

For the atom mapping questions, we explored two approaches of generating multiple SMILES strings: fully randomized and semicanonical, where SMILES were written by starting on a random atom but following canonical rules thereafter. When using semicanonical SMILES, the performance of o3-mini-high improved to 62%, compared to 50% with fully randomized SMILES (Table S2). This may be due to entire substructures of the molecule being traversed in the same order, yielding pairs of SMILES strings with subsequences of identical characters, thus allowing mapping of these atoms directly from the SMILES string without considering the molecular graph.

Overall, these results suggest that reasoning models may use heuristics to interpret SMILES strings, as opposed to parsing an entire molecular graph. Although the findings still demonstrate a basic understanding of SMILES notation, they show that reasoning models can not yet reliably parse SMILES strings and may struggle to interpret more complex molecular features that do not follow simple patterns.

### 3.5 Reasoning Models can Write IUPAC Names of Molecules

We then sought to test whether LLMs can map SMILES strings to chemical concepts. The most primitive requirement of this is to identify functional groups and describe their relationship to each other, which can be captured by the task of writing IUPAC names for molecules from SMILES strings. All previously tested models have achieved near-zero performance on IUPAC naming tasks.^18,19,29,30^

We found that reasoning-based LLMs are able to translate SMILES strings to IUPAC names for some molecules. The names generated by each model were generally not the “preferred IUPAC names” and could therefore only be evaluated by parsing them with the OPSIN tool and comparing the resulting structures to the ground truth molecule (Figure S8). The LLMs were initially tested using a sample of 100 molecules from the ZINC data set. On these questions, GPT-4o failed to generate any valid solutions, whereas o3-mini-low scored 10%, o3-mini-medium 18%, and o3-mini-high 29% (Figure 4). The best performing model was Gemini 2.5 Pro which scored 35–44% in the highest reasoning modes. In contrast to some other questions, the performance of o3-mini was similar when using randomized SMILES. There was a substantial overlap of molecules solved from both canonical and randomized SMILES, with o3-mini-high solving 16 molecules from both representations. These molecules are visualized in Figures S10 and S11.

**Figure 4:**
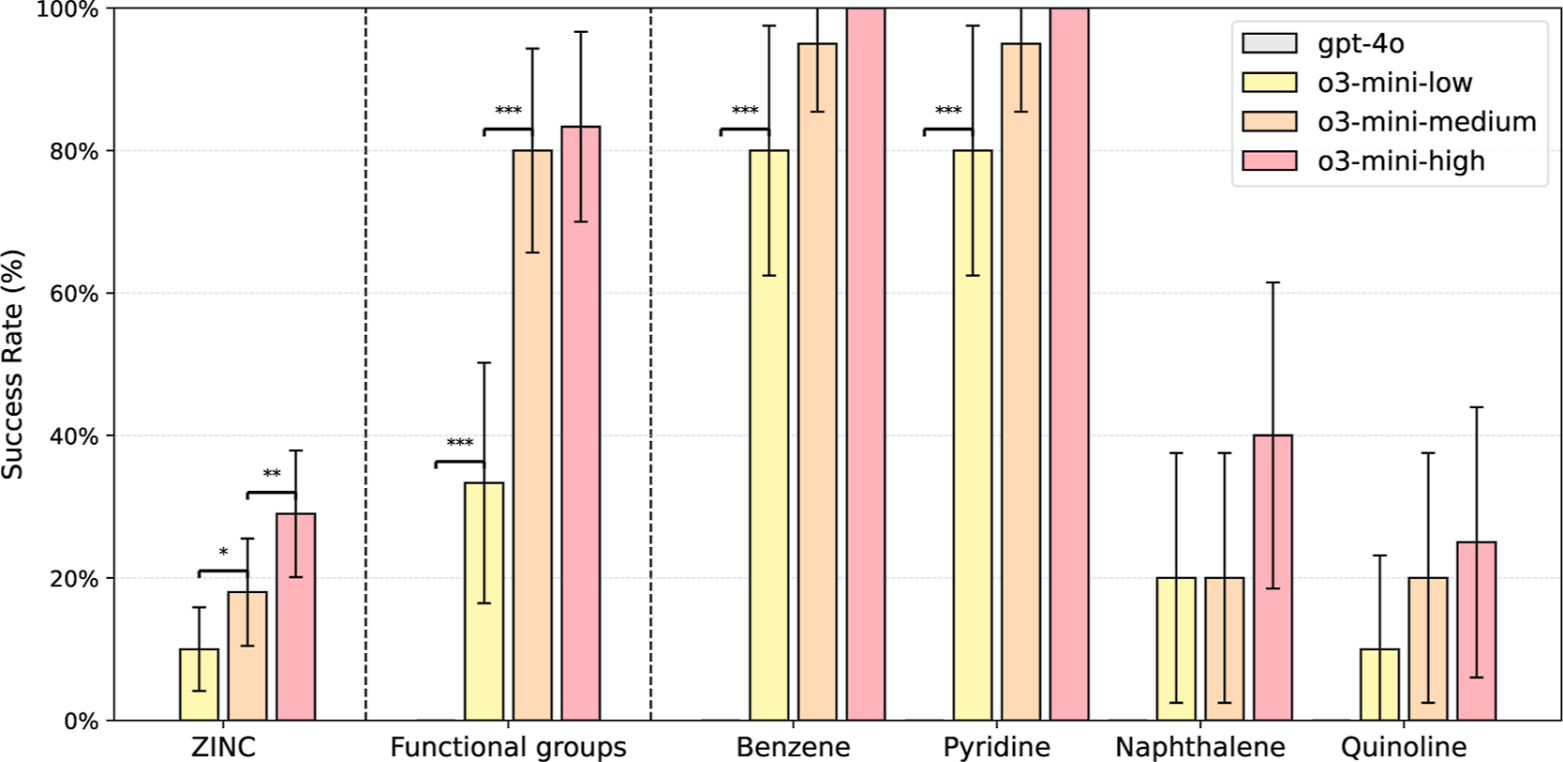
Performance on the SMILES to IUPAC questions. The o3-mini model can successfully write IUPAC names of some molecules. The GPT-4o model answered all questions incorrectly. ZINC molecules were sampled from the ZINC data set; correctly named molecules are shown in Figure S10. Functional group questions were constructed by attaching 6 different functional groups to a benzene scaffold (Figure S1). Locant numbering questions were constructed by attaching 3 halogens to the listed scaffolds (Figure S2). Names were accepted as correct if they could be parsed to the intended molecule by the OPSIN tool.^24^ Results shown are for canonical SMILES; see Table S2 and Table S10 for randomized SMILES. Vertical error bars show 95% confidence intervals (±1.96 × SE of the binomial proportion). Adjacent bars were compared using a one-tailed McNemar test. Significance levels are denoted as *p < 0.05, **p < 0.01, and ***p < 0.001.

Closer inspection suggested that the ability to write IUPAC names depended not only on molecular size, but also on the presence of specific substructures (Figure S7). For example, o3-mini-high’s naming accuracy dropped from 39.2% to 9.1% for molecules with a fused ring system. To further assess the main error modes on this question, we algorithmically generated two sets of molecules: one to test the ability to identify and name functional groups, and the other to test the ability to describe their positions within a molecule.

Using a set of 40 common functional groups, we randomly selected six groups and attached them at different positions on a benzene scaffold (example molecules are shown in Figure S1, further details are provided in Supporting Information B). o3-mini-low successfully produced IUPAC names for around 33% of these molecules, while the medium and high models both achieved ∼80% accuracy, demonstrating the models can accurately interpret functional groups from SMILES strings. We then tested the ability of the models to assign locant numbers by using a set of halogen-substituted scaffolds (Figure S2). o3-mini in all three reasoning modes could accurately number benzene and pyridine scaffolds with at least 80% success (Figure 4). The model often failed to number the fused ring systems of naphthalene and quinoline, scoring only ∼20% on these questions (Figure 4). This could be due to fused ring systems being more challenging to parse from SMILES and IUPAC locant numbering of fused systems being inherently more complex.

### 3.6 Reasoning Models Have the Potential to Interpret Chemical Trends

An important aspect of chemistry is the interpretation of chemical trends. We constructed a set of Free-Wilson analysis–type questions aimed at testing the ability to apply mathematical reasoning to a set of chemical structures. These questions were generated from additive linear equations and do not resemble real chemical trends. An example question is shown in Figure 5. All reasoning models achieved 95–100% on these questions. We also repeated these questions with the addition of noise to simulate more realistic data, and again, each reasoning model scored 95–100%. One common strategy used to solve these questions was via a system of equations (Table S17). These results suggest that the reasoning models are able to interpret mathematical relationships from molecular data and indicate that they may be able to analyze more complex experimental properties.

**Figure 5:**
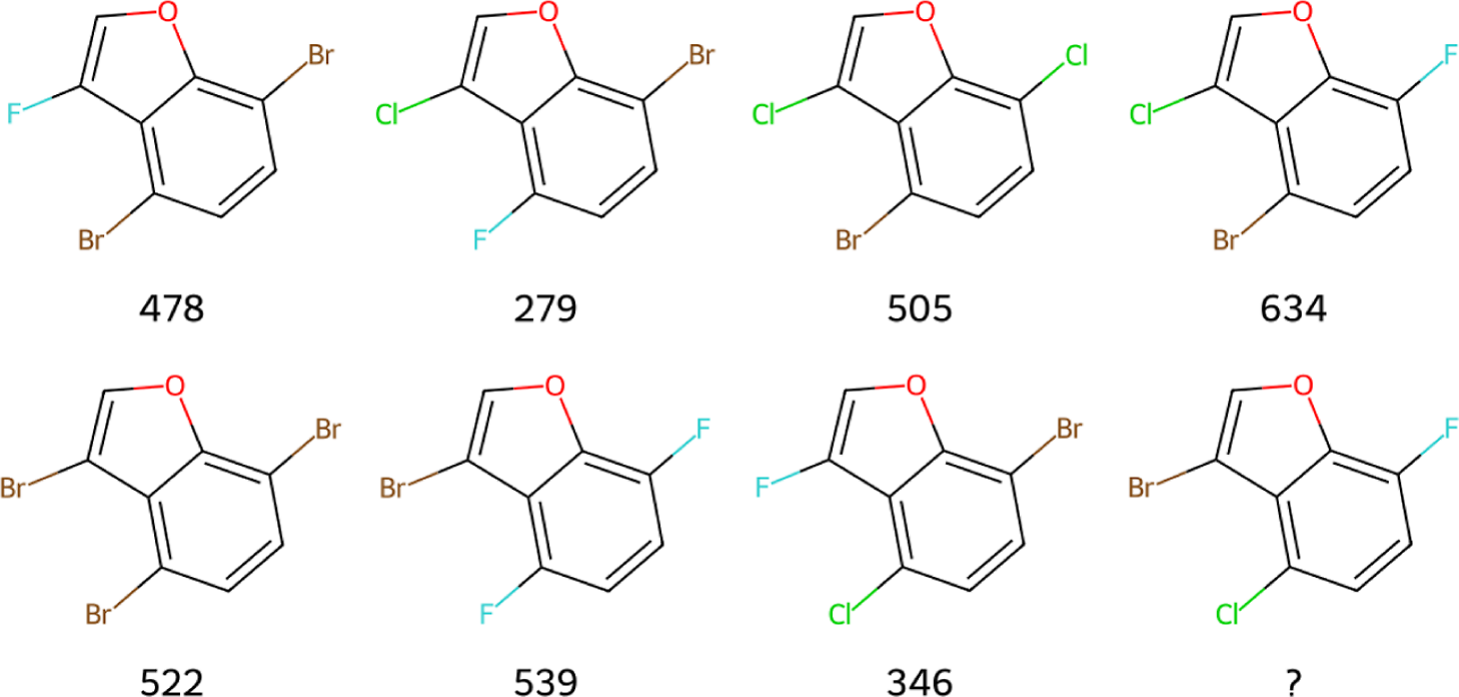
Example Free-Wilson analysis question. Molecules were constructed by attaching three halogens at three positions on a benzofuran scaffold. Each substituent at each position was assigned a random integer value, and the total score was calculated as the sum of these individual values. The task is to determine the score of the unknown molecule. The answer can be found in Table S17.

### 3.7 Reasoning Models can Write SMILES Strings for Simple Reactions

The ability to interpret and apply chemical concepts, and write SMILES strings, was assessed using a series of reaction prediction tasks. Nine undergraduate–level reaction classes were selected and five questions were generated for each. In each case, the model was prompted to write the product as a SMILES string. The chosen reactions varied in the complexity of the string manipulations required to convert the reactant SMILES to product SMILES. For instance, the SN2 questions require a simple substitution of Br to O, whereas the copper-catalyzed click reaction requires multiple bond orders to be changed, a ring formation, and the selection of the correct regioisomer.

All models demonstrated some degree of chemical understanding, with the reasoning models achieving significantly higher performance. For example, across all questions, GPT-4o scored 18%, o3-mini-low 36%, o3-mini-medium 51%, and o3-mini-high 56%. Performance by reaction class generally corresponded with the number of operations needed to write the final SMILES string. For instance, o3-mini-high achieved 100% accuracy on SN2 reactions, 50% on click reactions, and 18% on the Suzuki–Miyaura coupling (Figure S12). These results suggest that SMILES strings may still limit the expressive output of LLMs in chemistry tasks.

An example click-reaction question is shown in Table S18. In this task, the model was given two reactant SMILES strings and was prompted to write the product as a SMILES string. The corresponding reasoning excerpt ­(Figure 6) shows o3-mini-high using (1) SMILES strings, (2) IUPAC names, (3) structural formulas, (4) atom numbers, and (5) chemical language interchangeably to arrive at the answer, even though only SMILES notation was provided in the prompt. The excerpt in Table S18 also highlights a case in which the model initially generated an incorrect IUPAC name within its reasoning trace, then subsequently identified the error and corrected it.

**Figure 6:**
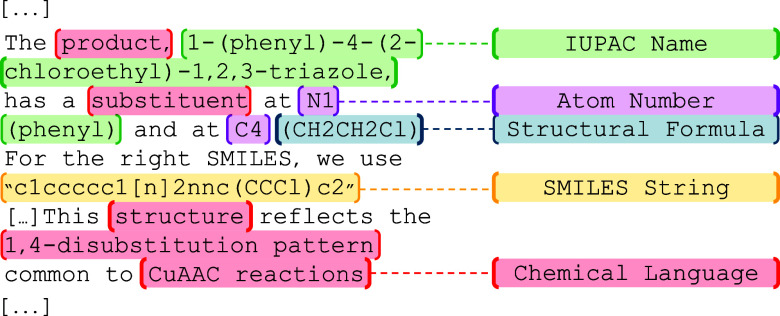
Annotated o3-mini-high reasoning excerpt for a reaction-prediction question. Highlighted text indicates distinct chemical representations used by the model. Similar combinations of representations are regularly observed in reasoning excerpts across all ChemIQ tasks. The full prompt and extended excerpt are provided in Table S18.

### 3.8 Structure Elucidation can be Solved by Reasoning Models

Finally, the most challenging question we tested the models with was NMR structure elucidation. Here, the models were provided with simulated ^1^H and ^13^C NMR data, along with a molecular formula, and were asked to write the corresponding molecular structure as a SMILES string. We began by testing the LLMs on molecules containing up to 10 heavy atoms from.^35^ The reasoning models solved the 1D NMR spectra of these molecules with 73–94% accuracy, compared with 20–30% accuracy of the nonreasoning models. The best performing model was Gemini 2.5 Pro which correctly determined the structures with 33–94% accuracy depending on the reasoning budget. When tested on 2D NMR of molecules sampled from ZINC, Gemini 2.5 Pro successfully solved ten out of 50 structures, including solving a structure consisting of 25 heavy atoms (Table 1). All 2D NMR molecules solved by Gemini 2.5 Pro at each reasoning budget are shown in Figure S13.

The reasoning excerpts for these questions show a resemblance to that of a human chemist. An example small molecule question is shown in Table S19. Here, o3-mini-high can be seen making a hypothesis that a carbon chemical shift corresponds to a carboxylic acid, before subsequently determining this to be inconsistent with the molecular formula. It then interprets methylene groups from the proton NMR and explains their deshielded chemical shift by proximity to an acetamide group. At this point, the model makes a comment that the integration of the proton peaks does not correspond to the number of protons given in the molecular formula, and proposes that the remaining protons must be exchangeable. Toward the end, the model proposes the IUPAC name of the structure, performs a double check, and then provides the correct SMILES string of the molecule.

The NMR elucidation excerpt from Gemini 2.5 Pro for the largest correctly solved ZINC molecule is given in Table 1 and shows advanced chemical reasoning. The model calculates the degree of unsaturation; identifies characteristic peaks; uses the HSQC to assign protons to specific carbon atoms; determines substructures using the COSY spectrum; combines substructures using the HMBC; focuses specifically on the HMBC of a CH_2_ group to connect two separate spin systems of the molecule; writes the IUPAC name of the molecule; then generates the final SMILES string. This result demonstrates a clear achievement in NMR elucidation and suggests that reasoning models can now, in some cases, address chemistry problems that previously relied on expert human judgment, marking a paradigm shift in computational chemistry.

### 3.9 Tool Augmentation Improves Model Performance

Prior work has demonstrated that LLMs can use external chemistry tools within agentic frameworks to solve multistep problems.^22^ More recently, reasoning models have been released that are capable of using tools as part of their reasoning process.^5^ To evaluate the effect of tool augmentation on model performance, we assessed OpenAI’s o4-mini model using the ChemIQ benchmark and medium reasoning effort, both with and without access to the “code interpreter” functionality. We observed an increase in performance from 51.7% to 65.7% when allowing tool access (Figure S16).

For the molecular interpretation tasks (carbon counting, ring counting, shortest path, and atom mapping), the model almost always used RDKit functions to directly answer the question (Figure S17). We also observed an improvement in the SMILES to IUPAC task from 17.5% to 27.5% with tool access, despite the absence of an IUPAC naming function in RDKit. Analyzing the code provided with the responses indicates that the model wrote code to iterate over atoms and output properties, such as atom index, symbol, and aromaticity, then used this information as additional context to answer the question. An example reasoning trace is given in Table S25, and an analysis of per question category tool calling is given in Figure S17. These results suggest that access to external tools can support intermediate reasoning steps, leading to improved performance on more complex tasks for which no specialized computational tools exists.

### 3.10 Model Limitations

Several limitations of the evaluated models beyond their quantitative benchmark performance were observed. The DeepSeek R1 model frequently failed to format its answer according to the given prompt. While most models occasionally suffered from this failure, this occurred significantly more frequently for DeepSeek R1. Implementing an algorithmic regular expression-based parser to extract the answer from the output significantly increased the performance of DeepSeek R1 from 24% to 50% accuracy, predominantly impacting the numerical and atom mapping questions (Table S4). Additionally, DeepSeek R1 exceeded its 32,000 token limit on 19 occasions, resulting in no generated response for these questions.

The OpenAI o3-mini-high model often encountered unspecified API errors, especially on the more challenging benchmark questions. We submitted four sets of ChemIQ questions in a single batch (3264 total prompts), and the initial response rate was 55.2%. Questions that failed due to API call errors were resubmitted up to two additional times, yielding a final response rate of 90.3%. The response rates by question category are reported in Table S22. Most failures occurred with the 2D NMR and atom mapping questions, which also had the highest reasoning token usage. We speculate that these errors may due to the model reaching its context window limit, rather than a technical API issue.

The Gemini 2.5 Pro model regularly exceeded the allocated reasoning budget. For example, despite an 8192-token allowance, the model used an average of approximately 12,000 reasoning tokens per response (Table S7).

## 4 Conclusion

Our results show that language models that have been explicitly trained to reason now possess the capability to directly solve advanced chemistry problems without using external tools or requiring prompt engineering. Specifically, across eight distinct tasks ranging from atom counting to NMR elucidation, o3-mini, Gemini 2.5 Pro, and DeepSeek R1 demonstrated the ability to interpret SMILES strings and reason on molecular graphs. For the first time, general-purpose LLMs were able to generate IUPAC names for molecules, with the reasoning models answering 29% to 44% of questions correctly when tested using the highest reasoning levels available. The performance of these reasoning models on the chemical reasoning tasks of NMR elucidation and reaction prediction signals a fundamental shift in the capabilities of LLMs. Our results strongly suggest that reasoning models now have the capacity to “think” about the structure of molecules, as opposed to just talking about them superficially. These findings represent a step change in the ability of language models to understand and reason about molecular structures.

Current reasoning models are still far from perfect. Their performance on the relatively simple carbon counting task, with only 74–92% accuracy, highlights limitations in basic SMILES parsing and interpretation that may contribute to errors across other tasks. All reasoning models were strongest at the Free-Wilson analysis (scoring 95–100%), which predominantly requires mathematical reasoning. We expect continued advances in the general capabilities of LLMs to improve performance on domain-specific benchmarks such as ChemIQ. Furthermore, we expect that domain-specific models tailored for chemical reasoning, as well as LLM-based systems augmented with external tools, will deliver significant performance gains over general-purpose LLMs.

In our experiments, we observed improved performance for higher levels of reasoning; it is not clear to what extent this trend would continue if current models were allowed to reason for longer. We observed a plateau in performance for Gemini 2.5 Pro at the highest reasoning budgets, suggesting that there may be a limit to the performance improvement from allowing current reasoning models to use additional reasoning tokens. However, this coincided with the model choosing not to use more tokens, rather than using more reasoning tokens ineffectively, and there was no such plateau for o3-mini based on the three reasoning levels available. Our results demonstrate that the generation of reasoning tokens is functionally useful for improving model performance; however, their correspondence to the model’s internal mechanisms remains uncertain. Further research is needed to verify the veracity of reasoning traces, both in chemistry and more broadly.

There are several potential weaknesses of ChemIQ. First, the risk of data leakage. Although it is possible that the molecules in our benchmark were present in the model pretraining data, our use of both canonical and randomized SMILES, together with tasks like shortest path and atom mapping, make it unlikely that the models have seen these specific questions before. Furthermore, in the SMILES to IUPAC naming tasks, the generated names were typically different from the preferred IUPAC names and could only be assessed using OPSIN. Taken together, this evidence supports the conclusion that these models are employing learned reasoning strategies rather than relying solely on memorization in order to answer these questions. Second, the questions in ChemIQ represent idealized tasks with unambiguous ground truth answers; evaluating model performance on more realistic problems involving experimental noise and incomplete data remains an important area for future work. Third, the prompts used in ChemIQ were designed to be simple, and a single prompt format was used for each question. Future work could investigate whether prompt and context engineering, such as using alternative molecular representations or providing examples (few-shot in-context learning), can further improve chemical reasoning. Finally, the aim of ChemIQ is to assess several prerequisites required for real-world chemical problem-solving. Currently, a number of the tasks are either trivial (e.g., counting carbons) or not routine (e.g., SMILES to IUPAC) for human chemists. Thus while a human baseline on ChemIQ would offer limited insight, it would be valuable in future benchmarks that assess more typical human tasks.

The same methodological advances enabling LLMs to solve complex chemistry problems risk lowering the barrier for producing chemical threats. The possibility that nonexperts could leverage these capabilities to produce chemical threats is a serious concern,^41^ and one that has been largely overlooked in recent safety assessments that have prioritized biological risks.^5,9^ It is essential that researchers in this field are proactive in addressing the safety and ethical implications of this technology to prevent its misuse. To address this, we recommend the development of chemistry-focused safety benchmarks that systematically test model behavior in threat creation, protocol optimization, and intent obfuscation. We further encourage community dialogue to establish clear safeguards, guidance, and norms for appropriate model deployment.

The emerging capabilities of LLMs point to a new paradigm where LLMs could serve as general-purpose chemical reasoning agents, combining formal theory and heuristic insights to solve a broad range of chemistry tasks. Such frameworks allow the integration of diverse information sources–such as patents, primary literature, and domain knowledge–into a single inference process, enabling context-aware decision-making and overcoming constraints that have historically limited conventional machine learning models. This could be transformational for data-limited chemical domains where developing task-specific models is currently often infeasible or insufficient. However, substantial improvements in accuracy, reliability, and molecular understanding are needed before this vision is realized.

## Supplementary Material



## Data Availability

All data (ChemIQ benchmark questions, answer checking scripts, and LLM outputs) for this study are available at https://github.com/oxpig/ChemIQ.

## Notes

Supporting Information (A) Chemistry benchmarks for LLMs (B) Methods (C) Results tables (D) Full prompts and reasoning excerpts (E) Additional analysis of ChemIQ results (F) Agentic model results (PDF)
